# Urinary tissue inhibitor of metalloproteinases-2 and insulin-like growth factor-binding protein 7 as early biomarkers of acute kidney injury and renal recovery following cardiac surgery

**DOI:** 10.1186/cc13571

**Published:** 2014-03-17

**Authors:** A Zarbock, M Meersch, C Schmidt, S Martens, J Rossaint, K Singbartl, D Görlich, J Kellum, H Van Aken

**Affiliations:** 1 University of Münster, Germany; 2 Penn State College of Medicine Hershey, PA, USA; 3 University of Pittsburgh, PA, USA

## Introduction

Difficulties in prediction and early identification of acute kidney injury (AKI) have hindered the ability to develop preventive and therapeutic measures for this syndrome. We tested the hypothesis that a urine test measuring insulin-like growth factor-binding protein 7 (IGFBP7) and tissue inhibitor of metalloproteinases-2 (TIMP-2), both inducers of G_1 _cell cycle arrest, a key mechanism implicated in AKI, could predict AKI in cardiac surgery patients.

## Methods

We studied 50 patients at high risk for AKI undergoing cardiac surgery with cardiopulmonary bypass. Serial urine samples were analyzed for [TIMP-2] × [IGFBP7] concentrations. The primary outcome measure was AKI as defined by international consensus criteria following surgery. Furthermore, we investigated whether urine [TIMP- 2] × [IGFBP7] could predict renal recovery from AKI prior to hospital discharge.

## Results

Twenty-six patients (52%) developed AKI. Diagnosis based on serum creatinine and/or oliguria did not occur until 1 to 3 days after cardiopulmonary bypass. In contrast, urine concentration of [TIMP-2] × [IGFBP7] rose from a mean of0.49 (0.24) at baseline to 1.51 (0.57) 4 hours after cardiopulmonary bypass in patients who developed AKI. The maximum urinary [T|MP-2] × [IGFBP7] concentration achieved in the first 24 hours following surgery (composite time point) demonstrated an area under the receiver-operating characteristic curve of 0.84. Sensitivity was 0.92, and specificity was 0.81 for a cutoff value of 0.50. The decline in urinary [TIMP-2] × [IGFBP7] values was the strongest predictor for renal recovery.

## Conclusion

Urinary [TIMP-2] × [IGFBP7] serves as a sensitive and specific biomarker to predict AKI early after cardiac surgery and to predict renal recovery.

